# Effects of exercise and diet in patients with incurable gastroesophageal cancer: the RADICES study

**DOI:** 10.1093/jncics/pkag006

**Published:** 2026-01-27

**Authors:** Aniek Bonhof, Anouk E Hiensch, Nicolette J Wierdsma, Linde F Huis In ‘t Veld, Sandra D Bakker, Aart Beeker, Marjan Davidis-van Schoonhoven, Helga Droogendijk, Jan C Drooger, Joeri A J Douma, Ruben S A Goedegebuure, Nadia Haj Mohammad, Irene E G van Hellemond, Karin Herbschleb, Johan J B Janssen, Bianca Mostert, Marije Slingerland, Dirkje Sommeijer, Liesbeth Timmermans, Arjan J Verschoor, Vincent A de Weger, Harm Westdorp, Miriam L Wumkes, Anne M May, Hanneke W M van Laarhoven

**Affiliations:** Julius Center for Health Sciences and Primary Care, University Medical Center Utrecht, Utrecht University, Utrecht, The Netherlands; Julius Center for Health Sciences and Primary Care, University Medical Center Utrecht, Utrecht University, Utrecht, The Netherlands; Department of Nutrition and Dietetics, Amsterdam University Medical Center, VU Medical Center, Amsterdam, The Netherlands; Cancer Center Amsterdam, Cancer Treatment and Quality of Life, Amsterdam, The Netherlands; Department of Medical Oncology, Amsterdam University Medical Center, University of Amsterdam, Amsterdam, The Netherlands; Department of Medical Oncology, Zaans Medical Center, Zaandam, The Netherlands; Department of Medical Oncology, Spaarne Gasthuis, Hoofddorp, The Netherlands; Department of Medical Oncology, Beatrix Hospital, Gorinchem, The Netherlands; Department of Internal Medicine, Bravis Hospital, Bergen op Zoom, The Netherlands; Department of Medical Oncology, Ikazia Hospital, Rotterdam, The Netherlands; Department of Medical Oncology, Frisius Medical Center, Leeuwarden, The Netherlands; Department of Internal Medicine, Meander Medical Center, Amersfoort, The Netherlands; Department of Medical Oncology, Imaging and Cancer, University Medical Center Utrecht, Utrecht University, Utrecht, The Netherlands; Department of Medical Oncology, Catharina Hospital, Eindhoven, The Netherlands; Department of Medical Oncology, Sint Antonius Hospital, Nieuwegein, The Netherlands; Department of Medical Oncology, Canisius Wilhelmina Hospital, Nijmegen, The Netherlands; Department of Medical Oncology, Erasmus Medical Center Cancer Institute, Erasmus University Medical Center, Rotterdam, The Netherlands; Department of Medical Oncology, Leiden University Medical Center, Leiden, The Netherlands; Department of Medical Oncology, Flevoziekenhuis, Almere, The Netherlands; Department of Primary and Community Care, Radboud University Medical Center, Nijmegen, The Netherlands; Department of Medical Oncology, Reinier de Graaf Gasthuis, Delft, The Netherlands; Department of Medical Oncology, Haga Hospital, Den Haag, The Netherlands; Department of Medical Oncology, Radboud University Medical Center, Nijmegen, The Netherlands; Department of Medical Oncology, Jeroen Bosch Hospital, Den Bosch, The Netherlands; Julius Center for Health Sciences and Primary Care, University Medical Center Utrecht, Utrecht University, Utrecht, The Netherlands; Cancer Center Amsterdam, Cancer Treatment and Quality of Life, Amsterdam, The Netherlands; Department of Medical Oncology, Amsterdam University Medical Center, University of Amsterdam, Amsterdam, The Netherlands

## Abstract

**Background:**

Patients with incurable gastroesophageal adenocarcinoma have an impaired health-related quality of life (HRQOL). Exercise combined with nutritional support may improve this outcome. Careful evaluation of this supportive care strategy is needed to avoid burdening patients at this vulnerable stage with interventions that may offer no (meaningful) benefit. Therefore, this study aims to investigate the effects of a combined exercise and nutritional intervention on HRQOL in patients with incurable gastroesophageal adenocarcinoma.

**Methods:**

RADICES (the effect of exeRcise And Diet on quality of life in patients with Incurable Cancer of Esophagus and Stomach) is a multicenter randomized controlled trial aiming to include 196 patients with incurable gastroesophageal adenocarcinoma. Participants are randomly assigned (1:1) to a patient-tailored intervention or a control group. The intervention group is provided with 2 training sessions per week and biweekly nutritional consultations, delivered by trained physiotherapists and dietitians, during 12 weeks. The control group receives usual care supplemented with general physical activity advice. The primary outcome is the difference in HRQOL between the intervention group and the control group at 12 weeks, accounting for baseline HRQOL, measured by the European Organization for Research and Treatment of Cancer Quality of Life Questionnaire–30 summary score. HRQOL is assessed at baseline, 6 weeks, 12 weeks, and every 3 months thereafter up to 1 year. Key secondary outcomes include patient-reported outcomes, cardiorespiratory fitness, dietary intake, disease progression, overall survival, and cost-effectiveness. Adherence and safety are monitored throughout the intervention period.

**Conclusion:**

This study will generate evidence on the effectiveness of a patient-tailored combined exercise and nutritional intervention in patients with incurable gastroesophageal adenocarcinoma. If effective for HRQOL, this intervention could be integrated into standard care for patients with incurable gastroesophageal adenocarcinoma.

**Trial registration:**

clinicaltrials.gov NCT06138223.

**Date of trial registration:** November 18, 2023

**Date and version study protocol:** 28-04-2025 version 3.1

**Date start recruitment:** 19-01-2024

## Introduction

For approximately 70% of patients diagnosed with gastroesophageal (adeno)carcinoma, curative treatment is not possible because of advanced disease and/or comorbidities.^1^ The survival of patients with incurable gastroesophageal adenocarcinoma is poor. For patients receiving best supportive care, survival is typically limited to a few months, whereas palliative systemic treatment can extend median survival to more than a year.^2-4^ In patients with incurable gastroesophageal adenocarcinoma, regardless of palliative treatment, health-related quality of life (HRQOL) is impaired.^5^^,^^6^ Across various cancer populations, potentially reversible factors contributing to this include reduced muscle mass, reduced physical capacity, and poor nutritional status.^7-9^

Exercise has been shown to positively affect multiple outcomes, including HRQOL and disease-free survival, during and after curative cancer treatment.^10-16^ In the metastatic breast cancer setting, a recent trial demonstrated beneficial exercise effects on HRQOL.^17^ Additionally, 2 systematic reviews support the safety and feasibility of exercise interventions in patients with different types of advanced cancer.^18^^,^^19^ However, specific evidence in patients with incurable gastroesophageal adenocarcinoma is lacking.^20^

Patients with incurable gastroesophageal adenocarcinoma are often malnourished,^21-24^ with reported prevalence rates between 80% and 96% in advanced stages of the disease, due to a combination of reduced appetite, impaired digestion and absorption, and metabolic changes.^25-29^ Moreover, the majority of these patients have a suboptimal energy and protein intake,^30-32^ although appropriate nutrition is essential for optimal exercise effects,^33^ and improvement of nutritional status has even been associated with increased survival.^34-36^

Although studies combining exercise and nutrition have been conducted, they mainly consist of pilot studies, take place in the curative setting, or consist of a population with different advanced cancer types.^14^^,^^37-41^ Two trials reported that a combined exercise and nutritional intervention is feasible (ie, attendance above 75%) and safe in patients with advanced cancer, including patients with incurable gastroesophageal adenocarcinoma.^37^^,^^38^ Also, improvements in handgrip strength,^38^ protein intake, and nausea and vomiting^37^ were observed in the exercise groups, but no effects on HRQOL could be detected, probably because of small sample sizes. More research is needed in incurable gastroesophageal adenocarcinoma patients to evaluate such a combined personalized intervention carefully,^20^ to avoid burdening patients at this vulnerable stage with interventions that may offer no meaningful benefit, and to ensure safety.

The RADICES (Effect of Exercise And Diet on Quality of Life in Patients with Incurable Cancer of Esophagus and Stomach) study has been primarily designed to investigate the effect of a 12-week combined exercise and nutritional intervention on HRQOL in patients with incurable gastroesophageal adenocarcinoma. Second, this study investigates, among others, the effects on other patient-reported outcomes, cardiorespiratory fitness, dietary intake, disease progression, overall survival, and cost-effectiveness. This article describes the design of the RADICES study.

## Methods

### Design

The RADICES study is a multicenter, randomized controlled trial with 2 study arms: (1) the intervention arm, which follows a 12-week patient-tailored combined supervised exercise and nutritional intervention in addition to usual care; and (2) the control arm, which receives a general physical activity and nutritional advice in addition to usual care. Both groups receive an activity tracker. The RADICES study protocol has been approved by the medical ethics committee of the Amsterdam University Medical Center (2023.0310—NL83835.018.23) in September 2023. Written informed consent will be obtained from all participants. The study is registered at clinicaltrials.gov under NCT06138223.^42^ The first patient was included on January 19, 2024. This study is being conducted according to the guidelines laid down in the Declaration of Helsinki.

### Participants

In the RADICES study, we aim to include 196 patients. To be eligible, patients need to meet the following inclusion criteria: incurable adenocarcinoma of esophagus or stomach with recurrence after treatment with curative intent or irresectable or metastatic disease at primary diagnosis, aged 18 years and older, able and willing to perform the exercise and nutritional intervention and wear an activity tracker, able and willing to fill out the questionnaires, and a life expectancy of at least 12 weeks as determined by the treating physician. Exclusion criteria are presence of unstable bone metastases inducing skeletal fragility, untreated symptomatic brain metastasis, serious active infection, being too physically active (ie, >210 min/week of moderate-to-vigorous exercise) or already engaging in an exercise program comparable to the RADICES exercise intervention, severe neurologic or cardiac impairment according to the American College of Sports Medicine criteria,^43^ uncontrolled severe respiratory insufficiency or being dependent on oxygen supplementation in rest or during exercise, uncontrolled severe pain, any other contraindications for exercise, pregnancy, and any (other) circumstances that would impede adherence to study requirements or ability to give informed consent. Medical inclusion and exclusion criteria are checked by the treating physician.

### Recruitment and random assignment

**Figure 1.**
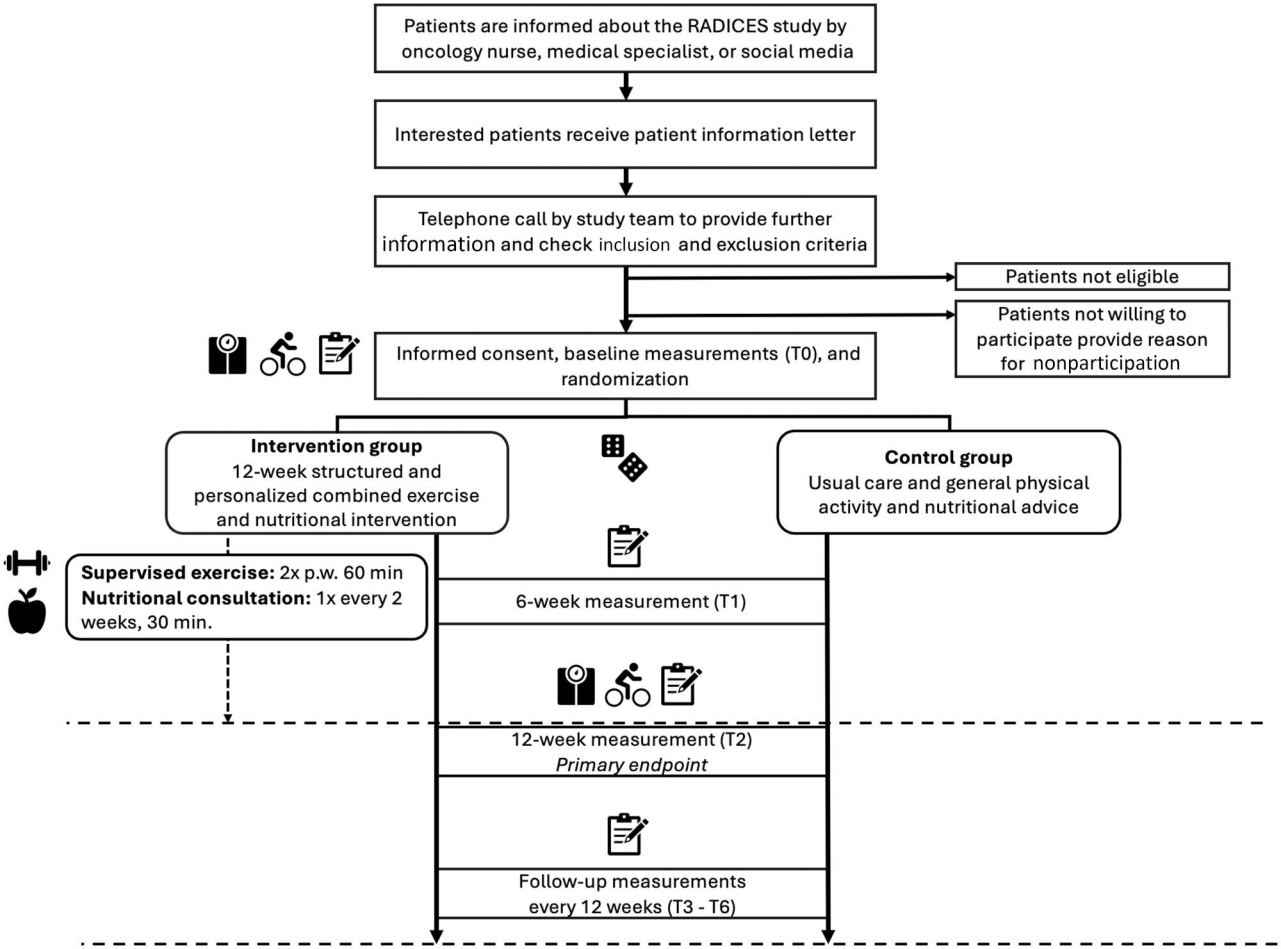
Recruitment and study procedures. Abbreviations: p.w. = per week; RADICES = effect of exeRcise And Diet on quality of life in patients with Incurable Cancer of Esophagus and Stomach.

Participants are being recruited from 22 hospitals across the Netherlands (Figure 1). Potentially eligible patients are informed about the study by the oncology nurse or medical specialist. Interested patients receive a patient information letter explaining the study aims and procedures. Subsequently, the patients are approached by the research team via telephone to provide further information, answer remaining questions, and verify inclusion and exclusion criteria. Patients who choose not to participate in the RADICES study are asked, but not required, to provide their reason for nonparticipation.

Eligible patients who are willing to participate are invited to the hospital to provide written informed consent and undergo baseline measurements. Hereafter, participants are randomly allocated (1:1) to either the combined exercise and nutritional intervention or the control group. All participants receive usual care according to the guidelines of their treating hospital, which generally includes systemic anticancer treatment and may include nutritional care if indicated. If hospitals refer patients to a physiotherapist who prescribes an exercise program comparable to the RADICES exercise intervention, these patients are not eligible for participation in our study. For the allocation procedure, a blocked computer-generated sequence (Castor EDC) is used, effectively blinding random assignment. Random assignment is stratified by the World Health Organization (WHO) performance status (0 vs 1 and higher) and treatment line (first vs second line or higher or best supportive care). Because of the nature of the intervention, participants, local medical staff, and investigators are not blinded to treatment assignment.

### Combined exercise and nutritional intervention

#### Exercise intervention

The exercise intervention consists of a moderate-to-high intensity 12-week supervised combined aerobic and resistance exercise program, with sessions lasting 1 hour, twice a week. The program is supervised by a trained (oncology) physiotherapist and is offered at a physical therapy practice located near the participants’ home address. Each participant has an intake session to tailor the standard exercise prescription to their individual needs. During the intervention, exercise intensity will be progressively increased and individually adjusted according to the participant’s current health status and perceived exertion, which are systematically assessed before, during, and after each exercise session. For a detailed description of the exercise program, see Supplementary Methods 1.

#### Nutritional intervention

The nutritional intervention consists of biweekly 30-minute consultations with a dedicated oncology dietitian. These consultations are held on-site or, if preferred by the participant, via telephone or video call. The dietitian will perform a nutritional assessment, make a dietetic diagnosis, set personalized nutritional goals, and provide individualized nutritional advice, taking into account treatment-related side effects (eg, nausea, taste changes), food preferences, and any alterations to the esophagus and stomach anatomy due to surgery.^28^ The purpose of the nutritional part of the intervention is to improve or maintain nutritional status by (1) preserving body weight (improvement of body weight when body mass index is less than 18.5 kg/m^2^ or when less than 20 kg/m^2^ if older than 65 years), (2) preserving or improving muscle mass and muscle strength, and (3) striving for sufficient energy and protein intake. Goals for energy and protein intake will be adjusted on an individual basis guided by monitoring body weight. The European Society for Clinical Nutrition and Metabolism guideline and the national guidelines of the National Nutritionists Oncology Working Group provide the means to reach this purpose.^44^^,^^45^ Additional details of the nutritional intervention are provided in Supplementary Methods 2.

### Control group

Participants in the control group receive usual care supplemented with general physical activity and nutritional advice according to current guidelines.^10^^,^^44^^,^^46^ During the 12-week intervention period, they are asked to maintain their usual physical activity pattern (ie, to not consult a physiotherapist if they had not done so before inclusion) and to avoid inactivity.

### Study outcomes

Participants visit the participating hospital for questionnaires and physical measurements at baseline and 12 weeks postbaseline (Table 1).

**Table 1.** Overview of all measurements in the RADICES study

Outcomes	Instrument	T0	T1	T2	Follow-up
		Baseline	6 weeks	12 weeks	Every 12 weeks
Primary outcome					
Health-related quality of life — summary score	EORTC QLQ-C30^47^	X	X	X	X
Secondary outcomes					
Patient-reported outcomes					
Quality of life functional and symptom scales	EORTC QLQ-C30^47^	X	X	X	X
Physical functioning, role functioning, fatigue	Computer adaptive testing	Every 2 weeks during intervention period			
Esophagogastric cancer specific symptoms	EORTC-QLQ-OG25^48^	X		X	X
Anxiety and depression	Hospital Anxiety and Depression Scale^49^	X		X	X
Physical measurements					
Resting heart rate and blood pressure	Blood pressure monitor	X		X	
Physical fitness	Steep Ramp Test, handgrip- and leg-strength test	X		X	
Anthropometry	Weighting scale (InBody Dial H20B)^50^	X		X	
Body mass index					
Body composition	Weighting scale (InBody Dial H20B)^50^, computed tomography scan	X		X	
Physical activity					
Self-reported (subjective)	Short questionnaire to assess health enhancing physical activity ^51^	X		X	X
Objectively measured physical activity	Physical activity tracker (Fitbit Inspire 2)	X		X	
Nutritional outcomes					
Malnutrition risk	Abridged Patient-Generated Subjective Global Assessment Short Form^52^	X		X	X
Sarcopenia risk	SARC-F^53^	X		X	X
Nutritional intake	Nutritional diary	X		X	
Cost-effectiveness					
QALY	EQ-5D-5L^54^	X		X	X
Productivity loss	Productivity Cost Questionnaire (IPCQ)^55^	X		X	X
Health-care resources consumption	Medical Consumption Questionnaire (iMCQ)^56^	X		X	X
Socio-demographic and medical data					
Sociodemographic data	POCOP questionnaire	X		X	X
World Health Organization performance status	Medical records	X		X	
Medical history and concomitant diseases	Medical records	X			
Cancer progress and treatment over the course of the study	Medical records	X		X	X
Cancer characteristics and treatment history	Medical records	X			
Concomitant medication	Medical records, interview	X		X	
Adverse events	Reports of patients, trainers, dietitians, oncology nurses, physicians, or medical records	X		X	
Toxicity and systemic treatment-related adverse events	Medical records	X	X	X	X
Overall survival and progression-free survival	Medical records and/or cancer registry	X	X	X	X
Intervention-specific outcomes					
Satisfaction with the intervention	Self-developed questionnaire			X	
Adherence	Recording session/consultation attendance			X	
Safety	Recording intervention-related (serious) adverse event	During the whole intervention period			

Abbreviations: EORTC-QLQ-C30 = European Organization for Research and Treatment of Cancer Quality of Life Questionnaire; EQ-5D-5L = EuroQol 5-Dimension 5-Level; iMCQ = iMTA Medical Cost Questionnaire; IPCQ = iMTA Productivity Cost Questionnaire; POCOP = Prospective Observational Cohort Study of Oesophageal-gastric Cancer Patients; RADICES = Effects of Exercise and Diet on Quality of Life in Patients with Incurable Cancer of Esophagus and Stomach; SARC-F = Strength, Ambulation, Rising from a Chair, Stair Climbing and History of Falling; SQUASH = Short questionnaire to assess health enhancing physical activity.

For those undergoing chemotherapy, assessments take place at least 3 days after chemotherapy infusion. Questionnaires are completed online or on paper at baseline, 6 weeks, and 12 weeks postbaseline. Additionally, every 2 weeks, patients receive a link via email to fill out computer adaptive testing questions. Subsequently, questionnaires are sent every 12 weeks up to 1 year postintervention. Questionnaires are sent through the Prospective Observational Cohort study of Oesophageal-gastric cancer Patients (POCOP) study, which is a prospective observational study for all Dutch patients with gastroesophageal cancer.^57^ After 1 year, participants will continue to be followed according to the POCOP study protocol. Furthermore, participants fill out a 3-day nutritional diary at baseline and at 12 weeks postbaseline. Personal data are coded, and all data are handled according to the General Data Protection Regulation (EU) 2016/679.

#### Primary outcome

The primary outcome of the RADICES study is HRQOL measured at 12 weeks using the European Organization for Research and Treatment of Cancer Quality of Life Questionnaire (EORTC QLQ-C30) summary score.^47^^,^^58^ This score includes all original QLQ-C30 subscales (ie, 5 functional scales [physical, role, emotional, cognitive, and social], 3 symptom scales [fatigue, nausea and vomiting, and pain], and 6 single items [dyspnea, insomnia, appetite loss, constipation, diarrhea, and financial difficulties]), except for the global HRQOL score and financial difficulties score. Scores range from 0 to 100, with a higher score indicating a better HRQOL.

### Secondary outcomes

Secondary outcomes include the EORTC QLQ-C30 functional and symptom subscales. In addition, physical functioning, role functioning, and fatigue subscales from the EORTC QLQ-C30 are assessed using computer adaptive testing every 2 weeks during the intervention period to improve measurement precision compared with the standard, static EORTC-QLQ-C30 questionnaire and to avoid floor- and ceiling-effects.^59^^,^^60^

Details on all other secondary outcome measures, as presented in Table 1, are described in detail in Supplementary Methods 3-13.

### Safety

All (serious) adverse events (related to the intervention or the study measurements are recorded by the study team, and (serious) adverse events are reported to the accredited ethical committee that approved the protocol.

### Sample size

No data on effect sizes on the EORTC QLQ-C30 summary score in patients with incurable gastroesophageal adenocarcinoma are available yet. Based on a systematic review of exercise trials, we based the sample size estimation on an effect size of 0.4.^61^ With 85 patients per group, an effect size of 0.40 can be detected with a power of 80% at a significance level of 5%, assuming a Pearson correlation coefficient between pre- and postintervention levels of Rho of 0.4. Taking repeated measurements into account using mixed models might further increase the study’s power. To account for a potential dropout rate of 15%, a total number of 196 patients will be enrolled in the study (*n* = 98 per study arm). The dropout rate will be reevaluated halfway, and the sample size will be recalculated if necessary to maintain adequate statistical power.

### Statistical analysis

Descriptive statistics will be used to characterize the study population at baseline. Questionnaire scores will be calculated according to published scoring manuals. Analyses will be performed according to the intention-to-treat principle.

For the primary outcome, mixed linear regression models will be used to assess intervention effects on HRQOL, while taking the hierarchical structure of the data into account. Models will be adjusted for the baseline value of the outcome and stratification factors (ie, WHO performance status and treatment line). In addition, the following covariates will be included in the model, if they improve the model fit: number of metastatic sites, HER2 status (negative or positive), programmed cell death ligand-1 status (Combined Positive Score < 5 or Combined Positive Score ≥ 5). The baseline table will be checked for disbalance in relevant factors (eg, first vs second line or beyond), and if necessary, we will correct for this in our analyses. The same analyses will be performed for secondary continuous outcomes.

Outcomes assessed at 2 time points (ie, physical fitness) will be analyzed as between-group differences in outcomes using analysis of covariance, adjusted for baseline values of the outcome and stratification factors. Dichotomous secondary outcomes (eg, doses reduction [yes, no]) will be assessed by logistic or Poisson regression models, also adjusted for stratification factors.

To assess the effect of the intervention on progression-free and overall survival, we will perform Cox proportional hazards models adjusted for stratification factors and, if necessary, for other prognostic factors.

We will perform mediation analysis to explore whether a potential effect of the intervention on the primary outcome can be explained by changes in, for example, physical capacity, muscle strength, muscle mass and fat mass, weight, nutritional status, or physical activity. Analyses for subgroups, for example, age, gender, and line of treatment will be performed by adding an interaction term to the corresponding models and stratified analyses.

Missing values of the patient-reported outcomes will be considered as missing at random and taken into account by using linear mixed-effects models. Reasons for missing primary endpoints will be critically assessed. If the number of missing primary outcome variables is high in both or 1 of the study groups, multiple imputation and other sensitivity analyses, such as using best- and worst-case scenarios, will be considered to explore potential biases. Because dropouts (ie, death or disease progression), and subsequently missing data, are often related to the HRQOL outcome (ie, informative dropout), a linear mixed model might lead to biased estimates. To avoid this, a joint model will be considered that consists of a linear mixed model for the longitudinal HRQOL outcome and a survival model for the time to dropout. The joint model will allow for the association between our primary outcome and time to dropout.

For the primary outcome, a 2-tailed *P* value less than .05 will be considered to indicate statistical significance. For all other outcomes, effect size estimates and 95% confidence intervals will be reported without *P* values. The widths of the confidence intervals will not be adjusted for multiple testing. For this reason, the intervals should not be used to derive definitive treatment effects for the secondary outcome or other outcomes.

Safety analyses will include tabulations of (serious) adverse events and χ^2^ tests. Furthermore, adherence to the intervention, contamination in the control group, and the number and reasons for dropout will be analyzed. Lastly, Supplementary Methods 14 describes the cost-effectiveness analysis.

### Data capturing and monitoring

Castor EDC, a cloud-based clinical data management platform, is used for random assignment and data capture. The POCOP registry is used to send questionnaires to patient-reported outcome measures to all participants.^57^ Validity of the data is checked by an independent monitor.

## Discussion

The aim of the RADICES study is to investigate the effect of a combined 12-week exercise and nutritional program on HRQOL in patients with incurable gastroesophageal adenocarcinoma.

Meta-analyses of exercise-oncology trials have suggested that supervised exercise programs, particularly those incorporating both aerobic and resistance training, are most effective.^11^ Building on our experience within the metastatic breast cancer setting, we set up a similar protocol in the current study.^62^ All exercise sessions are supervised by a trained physiotherapist to ensure safe execution of the exercise program. Given the expected high disease burden and fluctuating health in patients with incurable gastroesophageal adenocarcinoma, we anticipate that individual adaptations will be necessary. Thus, physiotherapists are instructed on how to select the appropriate exercise intensity and volumes,^17^^,^^46^^,^^62^ allowing adjustments in the prescribed exercise intensities based on the self-reported severity of exertion. We consider these adjustments as an appropriately tailored exercise prescription for this specific population rather than evidence of lack of feasibility or poor adherence. In addition, given the high risk of malnutrition, weight loss, and loss of muscle mass in this population, the exercise program is complemented by a tailored nutritional intervention provided by dedicated oncology dietitians to ensure adequate energy and protein intake and to support the maintenance of muscle mass. This approach is supported by findings from the Physical ExeRcise Following Oesophageal Cancer Treatment (PERFECT) trial in patients with resectable esophageal cancer, which showed that exercise improved HRQOL but was accompanied by involuntary weight loss and inadequate protein intake in the intervention group.^30^

There is ongoing debate about the amounts of energy and protein patients with cancer should minimally consume. According to the European Society for Clinical Nutrition and Metabolism guideline, the total energy expenditure of cancer patients can be assumed to be similar to that of healthy subjects, and observational studies in patients with esophageal and gastric cancer show similar results.^44^^,^^63^^,^^64^ However, it is well known that appropriate nutrition is vital for optimal training results, however, patients with incurable gastroesophageal adenocarcinoma are generally malnourished.^22-24^^,^^33^ Therefore, we advise an increased energy intake for illness and increased physical activity. Additionally, attention is given to sufficient protein intake and optimal timing of protein intake^44^^,^^45^^,^^65-70^ to prevent muscle protein breakdown and to enhance muscle protein synthesis. The nutritional intervention emphasizes practical dietary counseling to achieve energy and protein goals, including guidance on meal composition, portion sizes, and strategies to manage treatment-related side effects, enabling participants to implement the nutrition plan themselves in daily life.

Behavioral interventions can be challenged by low compliance to the intervention and contamination in the control group. To improve attendance and compliance, we offer the exercise sessions close to the patients’ homes, supervised by trained and experienced trainers. Moreover, the nutritional consultation may take place at the hospital or either via telephone or video calls depending on patient preference. Contamination (ie, the control group adopting something similar to the intervention) is reported to occur in 37% of exercise-oncology trials.^71^ We have implemented recommended measures to reduce contamination risk, such as including relatively inactive patients, clearly explaining the random assignment procedure, and the importance of the control group, as well as providing the control group with general exercise advice and an activity tracker. The activity tracker may provide a stimulus to engage in physical activity and has been shown to decrease dropout and contamination risk.^71^ Moreover, the activity tracker provides us with the opportunity to analyze objective measures of physical activity alongside subjective measures assessed by the SQUASH (short questionnaire to assess health enhancing physical activity) questionnaire.

During the first year of recruitment, we broadened our inclusion criteria from gastroesophageal adenocarcinoma patients receiving beyond first-line palliative treatment or best supportive care to any patient with recurrence or progression of gastroesophageal adenocarcinoma after curative treatment or irresectable or metastatic disease at diagnosis, regardless of timing or type of palliative treatment and number of treatment lines received. This was done to increase generalizability and offer the intervention earlier in the palliative phase to enhance its benefits. We acknowledge this will result in a more heterogeneous group and believe it will be more representative. Accordingly, we had to alter stratification. The first 23 patients were stratified by duration of first-line therapy (shorter or longer than 6 months), WHO performance status (0, 1, 2), and intended start of second-line systemic therapy (yes or no). After broadening the criteria, stratification factors were changed to WHO performance status (0 vs ≥1) and treatment line (first vs second line/higher/best supportive care). Findings in the metastatic breast cancer setting (unpublished results from PREFERABLE-EFFECT^17^) show no difference in intervention effects between treatment lines. Therefore, we assume that broadening this inclusion criterion will not negatively affect our expected effect size. However, we acknowledge that treatments and prognoses differ for patients with metastatic breast cancer or gastroesophageal adenocarcinoma, and we will investigate the effects by treatment lines.

In conclusion, the RADICES study investigates the effects of a combined supervised exercise and nutritional intervention on HRQOL, as well as other patient-reported, biomedical, and objective health outcomes in patients with incurable gastroesophageal adenocarcinoma. If our intervention is proven to be (cost-)effective, integrating exercise as a standard component of palliative care would be a logical next step. Additionally, our findings may contribute to international guidelines on the role of exercise and nutritional care improving HRQOL for patients with advanced disease.

## Supplementary Material

pkag006_Supplementary_Data

## Data Availability

No new data were generated or analyzed for this protocol paper.

## Participating Centers

Amsterdam University Medical Center, Bravis Hospital, Catharina Hospital Eindhoven, Canisius Wilhelmina Hospital, Diakonessenhuis, Erasmus Medical Center, Elisabeth Tweesteden Hospital, Flevoziekenhuis, Haga Hospital, Ikazia Hospital, Jeroen Bosch Hospital, Laurentius Hospital, Leids University Medical Center, Frisius Medical Center, Meander Medical Center, Radboud University Medical Center, Reinier de Graaf Hospital, Beatrix Hospital, Spaarne Gasthuis, Sint Antonius Hospital, University Medical Center Utrecht, Zaans Medical Center.
