# Supplementary material for: Effects of exercise and diet in patients with incurable gastroesophageal cancer: the RADICES study
Source: JNCI Cancer Spectr. 2026 Jan 27;10(2):pkag006. doi: 10.1093/jncics/pkag006 (PMC13021362; doi:10.1093/jncics/pkag006)
Supplement: pkag006_Supplementary_Data [file pkag006_supplementary_data.docx]

Supplementary Material

[Supplementary Methods 1: Detailed description exercise program 2](#_Toc213232289)

[Aerobic training 2](#_Toc213232290)

[Resistance training 2](#_Toc213232291)

[Unsupervised exercise program 3](#_Toc213232292)

[Supplementary Methods 2: Detailed description nutritional intervention 5](#_Toc213232293)

[Energy intake 5](#_Toc213232294)

[Protein intake 5](#_Toc213232295)

[Supplementary Methods 3: Secondary outcome measures: Esophagogastric cancer specific symptoms, anxiety and depression 6](#_Toc213232296)

[Esophagogastric cancer specific symptoms, anxiety and depression 6](#_Toc213232297)

[Supplementary Methods 4: Secondary outcome measures: Resting heart rate and blood pressure 7](#_Toc213232298)

[Resting heart rate and blood pressure 7](#_Toc213232299)

[Supplementary Methods 5: Secondary outcome measures: Physical fitness 9](#_Toc213232300)

[Aerobic capacity 9](#_Toc213232301)

[Muscle strength 9](#_Toc213232302)

[Supplementary Methods 6: Secondary outcome measures: Anthropometry and body composition 10](#_Toc213232303)

[Contraindications 10](#_Toc213232304)

[Precautions 10](#_Toc213232305)

[Measurement 11](#_Toc213232306)

[Disinfection 12](#_Toc213232307)

[Reporting 12](#_Toc213232308)

[Skeletal muscle mass analysis using CT scans 12](#_Toc213232309)

[Supplementary Methods 7: Secondary outcome measures: Physical activity 14](#_Toc213232310)

[Supplementary Methods 8: Secondary outcome measures: Nutritional status, malnutrition and dietary intake 15](#_Toc213232311)

[Supplementary Methods 9: Secondary outcome measures: Cost-effectiveness 16](#_Toc213232312)

[Supplementary Methods 10: Secondary outcome measures: WHO performance status 17](#_Toc213232313)

[Supplementary Methods 11: Secondary outcome measures: Systemic treatment toxicity, disease progression and survival 18](#_Toc213232314)

[Systemic treatment toxicity 18](#_Toc213232315)

[Disease progression and survival 18](#_Toc213232316)

[Supplementary Methods 12: Secondary outcome measures: Satisfaction with the intervention 19](#_Toc213232317)

[Supplementary Methods 13: Secondary outcome measures: Adherence 20](#_Toc213232318)

[Supplementary Methods 14: Statistical analysis: Cost-effectiveness 21](#_Toc213232319)

[Abbreviations 22](#_Toc213232320)

[References 24](#_Toc213232321)

# Supplementary Methods 1: Detailed description exercise program

## Aerobic training

The aerobic training consists of moderate-intensity continuous training (MICT) during the first 3 weeks, followed by high-intensity interval training (HIIT) for the remaining 9 weeks. The exercise intensity is gradually increased throughout the program (Table 1). The aerobic training protocol is individualized based on the patient’s baseline fitness level using the Maximal Short Exercise Capacity (MSEC) and estimated peak Wattage (W_peak_) as determined with the Steep Ramp Test (SRT; see Study Outcomes). For participants with bone metastases, aerobic training is preferably performed on a cycle ergometer or treadmill.

The perceived intensity of the aerobic training is assessed using the Borg scale. ^1^ Since this is a vulnerable population and health status may deteriorate, the exercise load should be reduced by 10% if the Rate of Perceived Exertion (RPE; 6-20) exceeds 15. The load can be increased by 10% if the RPE is below 13.

## Resistance training

The resistance training comprises 6 exercises targeting the major upper and lower body muscle groups. The exercises can be delivered using the following modalities: machine based, free-weights, or body weight. All resistance exercises are tailored to the patient’s fitness level by using 12-repetition maximum (12-RM) muscle tests as determined at the intake session at the physiotherapy practice.

Three sets per exercise are performed and the intensity is periodized (Table 1). To ensure a sufficient training load throughout the intervention period, the 12-RM muscle tests will be repeated every 4 weeks, with the resistance being adjusted accordingly (if the participant’s health status permits). When the weight used in a specific exercise is too high or low according to the participant or the judgment of the physiotherapist, the weight can be decreased or increased respectively by 5% or 10%. For participants with bone metastases, 12-RM testing is not performed for exercises that load regions with bone metastases (Table 2). ^2^ Adaptations to resistance exercises are shown in Table 2.

## Unsupervised exercise program

In addition to the supervised exercise program, participants are encouraged to be physically active for at least 30 minutes per day on all remaining days of the week. The participants are provided with an activity tracker (i.e., Fitbit Inspire 2). During the intake session, participants are supported by their physiotherapist to set appropriate and achievable exercise goals. Activities are documented and evaluated every 4 weeks with the physiotherapist, and adjustments are made if necessary.

*Table S1. RADICES exercise intervention protocol*

| Week | Aerobic training (15-30 min)^a^ | Resistance training (35 min) |
| --- | --- | --- |
| 1-3 | MICT at 50-60% of W_peak_ | Six exercises, with 3 sets per exercise^c^. The intensity is periodized, alternating between 10 to 12 reps at 70-75% of h1RM and 6 and 8 reps at 80-85% of h1RM every 4 weeks. |
| 4-8 | Interval training^b^: 8 x 1 min at W_peak_, alternated with 1 min active rest at 30 Watts |  |
| 9-12 | Interval training^b^: 3 x 3 min at 70% of W_peak_, alternated with 2 min active rest at 30 Watts |  |

*MICT Moderate-Intensity Continuous Training, MSEC Maximal Short Exercise Capacity, h1-RM hypothetical 1- Repetition Maximum*

*^a^ Since the prescribed training load applies to the cycle ergometer, this is the preferred machine for performing the aerobic training at least 1 of the 2 weekly sessions. The other training session can be performed on the rowing machine, treadmill, or elliptical/cross trainer.
^b^ All interval sessions start with a 3-minute warm-up at 30 Watts and conclude with a 3-minute cool-down.
^c^ Main exercises include the leg press, leg curl, leg extension, chest press, seated row, lateral pulldown. Variations of these exercises are allowed and depend on the exercise modality.*

*Table S2. Adaptations to the prescribed exercise program based on location of bone metastases*

| Site of metastases | Resistance exercise^a^ |  |  |  | Aerobic exercise |  |
| --- | --- | --- | --- | --- | --- | --- |
|  | Upper | Trunk | Lower |  | WB | NWB |
| Axial skeleton (thoracic/ribs) | *√^b^* |  | *√* |  | *√* | *√* |
| Axial skeleton (lumbar) | *√* |  | *√* |  |  | *√* |
| Pelvis | *√* | *√* | *√^c^* |  |  | *√* |
| Proximal humerus |  | *√^b^* | *√* |  | *√* | *√* |
| Proximal femur | *√* | *√* | *√^c^* |  |  | *√* |
| All regions | *√^b^* |  | *√^c^* |  |  | *√* |

*This table is adapted from Galvão et al (2011)* ^3^

*^a^ Resistance exercises that load the affected region can be either omitted according to this table or can be performed using a “start low, go slow” approach, depending on patient characteristics and the experience of the involved trainer. According to this approach, participants with bone metastases should start with low weights and more repetitions and increase weights gradually over time up to 10-12 repetitions if possible. Higher intensities (i.e., 6–8 repetitions with 80–85% of h1RM) should be avoided. Weights will be reduced if participants report pain during a resistance exercise or experience an increase in pain or new use or increase of pain medication since the last exercise session.*

*^b^ Exclusion of shoulder flexion/extension/abduction/adduction and inclusion of elbow flexion/extension*

*^c^ Exclusion of hip extension/flexion and inclusion of knee extension/flexion*

*√ = Target exercise region
WB weight bearing (e.g., walking), NWB non-weight bearing (e.g., cycling)*

# Supplementary Methods 2: Detailed description nutritional intervention

## Energy intake

The goal for all participants is to achieve a daily energy intake at the level of their estimated resting energy expenditure, calculated using the WHO equation for those with a BMI <30 kg/m^2^, and Harris-Benedict1919 equation for those with a BMI >30 kg/m^2^, increased by a factor (30-50%). for physical activity, illness and/or energy losses (e.g. vomiting, diarrhea) ^4,5^ On an individual basis, this initial goal may be adjusted based on ongoing monitoring of body weight.

## Protein intake

A protein intake of 1.5-1.9 g protein/kg fat free mass is recommended, based on body composition measurement using the InBody Dial H20B Smart Scales. ^6^ Additionally, participants are advised to consume a bolus of 15-25 grams of protein from their daily intake within 1-2 hours after exercise, to prevent muscle protein breakdown and to optimize the anabolic effect of protein for muscle protein synthesis. ^7-12^ The amount, type and timing of protein meals will be personalized.

When the energy and/or protein goals are not met through regular diet components, oral nutritional supplements, tube feeding, or in exceptional situations total parenteral nutrition may be added in accordance with the ESPEN guideline. ^13^

# Supplementary Methods 3: Secondary outcome measures: Esophagogastric cancer specific symptoms, anxiety and depression

## Esophagogastric cancer specific symptoms, anxiety and depression

To assess intervention effects on esophagogastric specific symptoms, not captured by the EORTC QLQ-C30, these symptoms are measured by the EORTC-QLQ-OG25, a validated 25-item esophagogastric module which contains questions on six symptom scales: dysphagia, eating restrictions, reflux, odynophagia, pain and anxiety. Scores range from 0 to 100, with higher scores indicating more symptoms. ^14^

Exercise is known to have positive effects on symptoms of anxiety and depression. ^15^ To evaluate this in patients with incurable GAC, symptoms of anxiety and depression are assessed using the Dutch version of the self-report Hospital Anxiety and Depression Scale (HADS) which contains two subscales: depression and anxiety. Each subscale ranges from 0 to 21, with higher scores indicating greater levels of depression or anxiety. ^16,17^

# Supplementary Methods 4: Secondary outcome measures: Resting heart rate and blood pressure

## Resting heart rate and blood pressure

Prior to the physical fitness and performance measurements, resting heart rate and blood pressure are measured to evaluate cardiovascular adaptations to the exercise and nutrition intervention and to ensure participant safety. All resting measurements are completed prior to the physical tests and participants should rest for at least 5 minutes prior to the resting measurements.

*Table S3. Blood pressure measurement*

| 1. Measurements can be taken in a sitting or supine position. |
| --- |
| 2. Measurements can be taken manually or with an electric blood pressure monitor. Record the device used on the form and use the same device for subsequent measurements. |
| 3. Instruct the participant not to talk during the measurement. |
| 4. Record the blood pressure on the form. |
| 5. Measurements must be taken twice and the measurement with the highest systolic pressure should be recorded. If the diastolic pressure is higher compared to the first measurement, but the systolic pressure is lower, record the blood pressure with the higher systolic pressure. |
| 6. If the participant has a PICC (peripherally inserted central catheter), blood pressure measurements should not be taken on that arm. |
| 7. If the participant has high-normal blood pressure (systolic 120-129 mmHg and/or diastolic 80-89 mmHg) or hypertension (systole >130 mmHg and/or diastole >90 mmHg), wait 1 minute and repeat the measurement. Record the lower value on the form. |
| 8. Participants with severe hypertension at rest (systolic blood pressure >180 mmHg and/or diastolic blood pressure >110 mmHg) should be referred to an internist or cardiologist (depending on local protocols and procedures). Physical tests should not be performed by these patients. |

*Table S4. Heart rate measurement*

| 1. Measurements can be taken in a sitting or supine position. |
| --- |
| 2.a) When using an electric blood pressure monitor, the heart rate can be read from the device.  2.b) When measuring blood pressure manually, the heart rate must be measured separately using a heart rate belt. Then write down the heart rate displayed after one minute.  It may be necessary to apply some water to the inner surface of the chest strap to improve signal conductivity before applying the Polar heart rate belt. |
| 3. Write down the heart rate on the form. |

# Supplementary Methods 5: Secondary outcome measures: Physical fitness

Physical fitness measurements include aerobic capacity and upper- and lower body muscle strength.

## Aerobic capacity

To quantify cardiovascular fitness, which is expected, in the current setting, to be maintained or improve through exercise and contributes to physical functioning, we measure aerobic capacity. This ismeasured as the MSEC using the SRT, performed by a trained local researcher. ^18-20^ After 3 minutes of unloaded cycling, the test starts at 25 Watts and is increased by 2.5 Watts per second or 25 Watts per 10 seconds until exhaustion. Participants are instructed to cycle with a pedal frequency between 70 and 90 rpm. The test ends when pedal frequency falls below 60 rpm, or when the participant experiences any pain or anxiety. After termination, the participant is asked to continue cycling at an easy cadence with minimal load to recover. The outcome is noted as the highest achieved output in Watts and is referred to as MSEC. From the MSEC, W_peak_ can be estimated using a regression equation.^18^ Additionally, RPE at termination, time cycled and heart rate directly after termination and 1 and 2 minutes later is recorded.

## Muscle strength

We measure muscle strength to assess improvements in muscular function and strength as a direct effect of exercise and as a proxy for survival, which is low in our population. Muscle strength is assessed by handgrip strength and by leg muscle strength. Handgrip strength is assessed using a handgrip dynamometer (hydraulic Jamar®). The participant is asked to squeeze the dynamometer as hard as possible. Three measurements are performed on both hands. The best attempt is recorded for each hand. Leg strength is assessed using a leg press hypothetical 1-RM test (h1-RM) according to a standardized protocol, unless there are bone metastases that prohibit safe testing. For the h1-RM test, the highest weight that was successfully lifted for 12 repetitions and the corresponding h1-RM is recorded. ^21^

# Supplementary Methods 6: Secondary outcome measures: Anthropometry and body composition

Weight, fat mass (in %) and Muscle Mass (in kg) are measured with the InBody Dial H20B ^6^ Smart Scales prior to the physical fitness tests. Weight and BIA measurement are important baseline measures to be able to individualize the intervention and set personalized goals. It is also measured to evaluate changes in body composition reflecting the combined impact of exercise and nutritional support on nutritional status.

Contraindications
- Pacemaker
When screening for inclusion criteria of the RADICES study, the doctor determined that there is no electrical implant. Check and ask the participant whether it is true that the participant does not have an electrical implant; If the participant has a pacemaker, measure the participant on a regular scale and contact the study team.

- Pregnancy
When screening for inclusion criteria of the RADICES study, it was asked whether there is no pregnancy. Check and ask the participant if this is still the case, if applicable.

Precautions
In order to obtain accurate results of the measurement, the researchers must instruct the participant to:
- Not perform any intensive physical activity 24 hours before the measurement;
- Not visit a sauna or take a hot shower 24 hours before the measurement;
- Come to the test with an empty bladder. Ask the participant to empty it if the bladder is full. The test will be performed even if the above points are not met. This will be noted on the form.

In addition, it is important to check the following points before the measurement and to note them on the form:
- Burns or decubitus;
- Abnormal hydration status (e.g. oedema, ascites, dehydration, dialysis, medication that causes fluid loss or retention);
- Menstruation.

Before the measurement, ensure that the participant:
- Wears no accessories, has empty pockets, or is not wearing heavy clothing.

Try to measure under consistent measurement conditions ((body) temperature, time of day, time of week, etc.).

N.B. Although the current guideline of the European Society for Clinical Nutrition and Metabolism (ESPEN) and the InBody manual advise to perform BIA measurements in a fasting state, with at least 2 hours recommended in a clinical setting and 8 hours in a research setting, the participant does not have to fast for the BIA measurement in the context of the RADICES study. It is undesirable to ask patients with (increased risk of) malnutrition and/or sarcopenia to fast and there is no such literature that supports this strategy in the guideline, according to the SOP of the Nutritional Assessment Platform. ^22^

Measurement
Moisten the electrodes with water and switch on the InBody (press the round button).

To obtain reliable results, enter the accurate length of the patient in the device. Turn the knob to enter the length. Instruct the participant to stand on the InBody with bare feet.

Note:
- Heels on rear electrodes;
- Arms stretched;
- Thumbs on thumb electrodes, thumbs do not touch.

Turn the round knob to view the results. Note the results of weight, fat percentage and muscle mass.

Disinfection
Clean the scale with alcohol.

Note! Do not use disinfectant that contains hydrogen peroxide because it may damage the electrodes.

Reporting
Note weight (kg), fat percentage (%), muscle mass (kg) and visceral fat level.

In case of a measurement on a regular scale because the participant has a pacemaker, note the weight on the form and tick that was measured on a regular scale.

## Skeletal muscle mass analysis using CT scans

To provide a precise and validated measure of muscle mass in addition to our BIA measurements, body composition is also assessed by making use of CT-scan images of patients receiving palliative systemic treatment to assess systemic treatment efficacy. These CT-scans are planned and made according to standard care.

Using a deep learning based and semi-automatic tool, muscle mass will be quantified by Skeletal Muscle Area (SMA) measurements. Subsequent Skeletal Muscle Index (SMI; SMA/H^2^ in cm^2^/m^2^) will be calculated. Skeletal muscle and fat compartments will be demarcated using pre-determined cut-off points for Houndsfield units (-29 to +150) and for low muscle mass according to current guidelines. ^23-25^ Muscle quality (i.e. myosteatosis) will be quantified as Muscle Radiation Attenuation (MRA). The MRA for skeletal muscle is assessed by calculating the average HU value of the total MRA. CT-scans dated closest to the baseline and 12-week post-baseline measurements will be used for analyses. CT-scans will be excluded if they are not suitable for muscle analysis (e.g. L3 level not fully present on the scan, presence of artefacts, or insufficient scan quality due to low resolution or scattering).

# Supplementary Methods 7: Secondary outcome measures: Physical activity

To allow for assessment of contamination, we use a subjective and objective measure. Self-reported physical activity is assessed using the validated and reliable Short QUestionnaire to ASsess Health enhancing physical activity (SQUASH) including commuting activities, leisure time activities, household activities, and activities at work and school. ^26^

Additionally, an activity tracker (Fitbit Inspire 2) is used to objectively measure step count, heart rate and physical activity minutes. Participants from both groups are instructed to wear the tracker throughout the whole intervention period, but in any case, seven days after randomization and the week before the 12-week post-baseline assessments. Mean daily steps and minutes of different intensity levels of physical activity will be calculated, excluding non-wear days.

# Supplementary Methods 8: Secondary outcome measures: Nutritional status, malnutrition and dietary intake

To screen for sarcopenia and malnutrition risk, and to evaluate whether the intervention improves these risks, nutritional status is screened with multiple tools. Malnutrition is screened using the abridged scored Patient-Generated Subjective Global Assessment (abPG-SGA), developed for cancer patients in an outpatient setting. ^27^ Risk of sarcopenia is screened by the Strength, Ambulation, Rising from a Chair, Stair Climbing and History of Falling (SARC-F) questionnaire. Sarcopenia will be assessed by determining muscle mass (CT or InBody) and handgrip strength according to criteria from the European Working Group on Sarcopenia in Older People 2 (EWGSOP2) ^28-30^. Furthermore, the Global Leadership Initiative on Malnutrition (GLIM) criteria will be used for further assessment of malnutrition. For phenotypic criteria BMI, information from the abPG-SGA on weight loss, and information from the bioimpedance analysis (BIA) measurement and CT-scans on muscle mass will be used. For etiologic criteria information on reduced food intake, derived from the abPG-SGA, or information on inflammation as defined by the presence of acute disease or injury, or chronic disease, will be used. ^31^ Since patients with advanced cancer usually have high CRP levels, the assumption will be made that the etiological component of GLIM, defined as ‘chronic disease-related inflammation’, is met in our patient sample.

During follow-up, risk of malnutrition and sarcopenia will be re-assessed by the SARC-F and abPG-SGA questionnaire, every three months.

Changes in energy and protein intake will be estimated using a nutritional diary for three days, at baseline and at 12 weeks post-baseline. Researchers from the Amsterdam UMC and UMC Utrecht assess completeness shortly after receiving the diary and if information is missing, participants are contacted to provide additional information. The content of the diaries is entered in ‘Mijn Eetmeter’, an application from the government-subsidised Nutrition Centre of the Netherlands ^32^, to calculate total energy- and protein intake per day.

# Supplementary Methods 9: Secondary outcome measures: Cost-effectiveness

To evaluate cost-effectiveness of the intervention, multiple questionnaires are used. The EuroQol 5-Dimension 5-Level (EQ-5D-5L) is used to measure health in five dimensions, including mobility, self-care, usual activities, pain/discomfort, and anxiety/depression, using 5 levels of severity. ^33^ This questionnaire is used to calculate quality adjusted life years (QALYs) during follow-up. The actual costs associated with both strategies (intervention versus control) will be compared up until one year after randomization. The cost-effectiveness analysis will be performed from a societal perspective, including healthcare costs, patient and family costs and productivity costs. Participants will complete questionnaires to collect data on these different types of costs.

A healthcare use questionnaire was developed based on the iMTA Medical Cost Questionnaire (iMCQ), including cost categories that are deemed relevant for patients with GAC. ^34^ Productivity losses are measured using the Productivity Cost Questionnaire (iPCQ). ^35^

# Supplementary Methods 10: Secondary outcome measures: WHO performance status

WHO performance status is assessed by the research nurse or treating physician at baseline, at 12 weeks and before each treatment cycle during the intervention period. ^36^ It serves as a stratification factor. We also measure WHO performance status to assess overall functional status using a simple and widely used clinical scale.

# Supplementary Methods 11: Secondary outcome measures: Systemic treatment toxicity, disease progression and survival

## Systemic treatment toxicity

To assess whether the intervention influences treatment tolerance or the occurrence of adverse events, start and stop dates of systemic palliative therapy and type of treatment are recorded. Additionally, adverse events during systemic treatment are assessed according to the Common Terminology Criteria for Adverse Events (CTCAE) version 5.0. ^37^ Moreover, dose reductions and delays due to toxicity are recorded. If a participant switches lines, only information on the first line received within the study period will be recorded.

## Disease progression and survival

Participants will be followed for disease progression and survival up to 1 year after inclusion of the last patient in the study, in order to assess whether the intervention influences disease progression and survival. Information on disease progression and (all-cause and GAC specific) death is retrieved from medical records and/or the cancer registry. Progression is defined as the first day when the Response Evaluation Criteria In Solid Tumors (RECIST) version 1.1 criteria for progressive disease are met, ^38^ or in case of non-measurable disease, based on clinical progression.

# Supplementary Methods 12: Secondary outcome measures: Satisfaction with the intervention

At 12 weeks post-baseline, satisfaction with the combined intervention is assessed by means of a self-designed questionnaire, containing questions regarding the supervised exercise program, the trainer, the activity tracker, the nutritional program and the dietitian. Evaluation of this questionnaire enables us to assess participants’ acceptability and perceived value of the program for future implementation.

# Supplementary Methods 13: Secondary outcome measures: Adherence

Adherence to the exercise intervention will be evaluated by recording session attendance and adherence to the prescribed exercise intensity. Attendance and compliance are recorded by the physiotherapist in an electronic case report form. Adherence to the nutritional intervention will be evaluated by recording consult attendance and percentage of achieved intake of prescribed calories and protein, both recorded by the dietitian in an electronic case report form. This data will be collected to quantify attendance to and compliance with the exercise and nutritional intervention.

# Supplementary Methods 14: Statistical analysis: Cost-effectiveness

In the economic evaluation, the balance between costs and effects of both study arms will be assessed and compared up until 1-year post-baseline. Results of both cost and effect measurement will be integrated using cost-effectiveness and cost-utility analyses. In the cost-utility analysis, efficiency is expressed in terms of costs per QALY. In the cost-effectiveness analysis, costs per unit of change in the primary outcome measure will be estimated. Finally, incremental costs and incremental effects, expressed in a ratio (ICER) will be estimated. Deterministic and probabilistic sensitivity analysis will be applied, using bootstrapping techniques.

# Abbreviations

abPGS-GA abridged scored Patient-Generated Subjective Global
 Assessment

BIA Bioimpedance Analysis

CTCAE Common Terminology Criteria for Adverse Events

EORTC-QLQ-OG25 European Organization for Research and Treatment of
 Cancer Quality of Life for Oesophageal and Gastric
 symptoms

ESPEN European Society for Clinical Nutrition and Metabolism

EWGSOP2 European Working Group on Sarcopenia in Older People 2

EQ-5D-5L EuroQol 5-Dimension 5-Level

GAC Gastroesophageal adenocarcinoma

GLIM Global Leadership Initiative on Malnutrition

HADS Hospital Anxiety and Depression Scale

HIIT High-intensity interval training

HRQoL Health-related quality of life

ICER Incremental costs and incremental effects ratio

iMCQ iMTA Medical Cost Questionnaire

IPCQ iMTA Productivity Cost Questionnaire

LWDO Landelijke Werkgroep Diëtisten Oncologie

MICT Moderate-intensity continuous training

MRA Muscle Radiation Attenuation

MSEC Maximal Short Exercise Capacity

NWB non-weight bearing

QALY Quality Adjusted Life Year

RECIST Response Evaluation Criteria In Solid Tumors

RM Repetition Maximum

H1-RM Hypothetical 1 Repetition Maximum

SARC-F Strength, Ambulation, Rising from a Chair, Stair Climbing
 and History of Falling

SMA Skeletal Muscle Area

SMI Skeletal Muscle Index

SRT Steep Ramp Test

SQUASH Short questionnaire to assess health enhancing physical
 activity

WB Weight-bearing

WHO World Health Organization

Wpeak peak Wattage

# References

1. Borg GA. Psychophysical bases of perceived exertion. *Med Sci Sports Exerc.* 1982;14(5):377–381

2. Campbell KL, Cormie P, Weller S, et al. Exercise Recommendation for People With Bone Metastases: Expert Consensus for Health Care Providers and Exercise Professionals. *JCO Oncol Pract.* 2022;18(5):e697–e709

3. Galvão DA, Taaffe DR, Cormie P, et al. Efficacy and safety of a modular multi-modal exercise program in prostate cancer patients with bone metastases: a randomized controlled trial. *BMC Cancer.* 2011;11:517

4. Harris JA, Benedict FG. A Biometric Study of Human Basal Metabolism. *Proc Natl Acad Sci U S A.* 1918;4(12):370–373

5. Kruizenga HM, Hofsteenge GH, Weijs PJM. Predicting resting energy expenditure in underweight, normal weight, overweight, and obese adult hospital patients. *Nutr Metab (Lond).* 2016;13:85

6. InBody Co., Ltd. (n.d.), Seoul, Korea. InBody Dial H20B.<https://inbody.com/en/homeuse/contents/view_dial>

7. Hoffer LJ, Bistrian BR. Appropriate protein provision in critical illness: a systematic and narrative review. *Am J Clin Nutr.* 2012;96(3):591–600

8. Churchward-Venne TA, Holwerda AM, Phillips SM, van Loon LJC. What is the Optimal Amount of Protein to Support Post-Exercise Skeletal Muscle Reconditioning in the Older Adult? *Sports Med.* 2016;46(9):1205–1212

9. Pearson AG, Hind K, Macnaughton LS. The impact of dietary protein supplementation on recovery from resistance exercise-induced muscle damage: A systematic review with meta-analysis. *Eur J Clin Nutr.* 2023;77(8):767–783

10. Trommelen J, van Lieshout GAA, Pabla P, et al. Pre-sleep Protein Ingestion Increases Mitochondrial Protein Synthesis Rates During Overnight Recovery from Endurance Exercise: A Randomized Controlled Trial. *Sports Med.* 2023;53(7):1445–1455

11. Wall BT, Gorissen SH, Pennings B, et al. Aging Is Accompanied by a Blunted Muscle Protein Synthetic Response to Protein Ingestion. *PLoS One.* 2015;10(11):e0140903

12. Moore DR, Churchward-Venne TA, Witard O, et al. Protein ingestion to stimulate myofibrillar protein synthesis requires greater relative protein intakes in healthy older versus younger men. *J Gerontol A Biol Sci Med Sci.* 2015;70(1):57–62

13. Arends J, Bachmann P, Baracos V, et al. ESPEN guidelines on nutrition in cancer patients. *Clin Nutr.* 2017;36(1):11–48

14. Lagergren P, Fayers P, Conroy T, et al. Clinical and psychometric validation of a questionnaire module, the EORTC QLQ-OG25, to assess health-related quality of life in patients with cancer of the oesophagus, the oesophago-gastric junction and the stomach. *Eur J Cancer.* 2007;43(14):2066–2073

15. Campbell KL, Winters-Stone K, Wiskemann J, et al. Exercise Guidelines for Cancer Survivors: Consensus statement from International Multidisciplinary Roundtable. *Med Sci Sports Exerc.* 2019;51(11):2375–2390

16. Zigmond AS, Snaith RP. The hospital anxiety and depression scale. *Acta Psychiatr Scand.* 1983;67(6):361–370

17. Spinhoven P, Ormel J, Sloekers PP, Kempen GI, Speckens AE, Van Hemert AM. A validation study of the Hospital Anxiety and Depression Scale (HADS) in different groups of Dutch subjects. *Psychol Med.* 1997;27(2):363–370

18. Stuiver MM, Kampshoff CS, Persoon S, et al. Validation and Refinement of Prediction Models to Estimate Exercise Capacity in Cancer Survivors Using the Steep Ramp Test. *Arch Phys Med Rehabil.* 2017;98(11):2167–2173

19. De Backer IC, Schep G, Hoogeveen A, Vreugdenhil G, Kester AD, van Breda E. Exercise testing and training in a cancer rehabilitation program: the advantage of the steep ramp test. *Arch Phys Med Rehabil.* 2007;88(5):610–616

20. Morton RW, Traylor DA, Weijs PJM, Phillips SM. Defining anabolic resistance: implications for delivery of clinical care nutrition. *Current Opinion in Critical Care.* 2018;24(2):124

21. J. Landers. Maximums based on reps. *Natl Strength Cond Assoc J.* 1984;6(6):60–0

22. Nutritional Assessment Platform. Standard Operating Procedure BIA. <https://nutritionalassessment.nl/> Web site. <https://nutritionalassessment.nl/standaard-operating-procedure-bia/>. Accessed 19-08, 2025

23. Prado CMM, Lieffers JR, McCargar LJ, et al. Prevalence and clinical implications of sarcopenic obesity in patients with solid tumours of the respiratory and gastrointestinal tracts: a population-based study. *Lancet Oncol.* 2008;9(7):629–635

24. van Vugt JLA, Levolger S, Gharbharan A, et al. A comparative study of software programmes for cross-sectional skeletal muscle and adipose tissue measurements on abdominal computed tomography scans of rectal cancer patients. *J Cachexia Sarcopenia Muscle.* 2017;8(2):285–297

25. Dijksterhuis WPM, Pruijt MJ, van der Woude SO, et al. Association between body composition, survival, and toxicity in advanced esophagogastric cancer patients receiving palliative chemotherapy. *J Cachexia Sarcopenia Muscle.* 2019;10(1):199–206

26. Wendel-Vos GCW, Schuit AJ, Saris WHM, Kromhout D. Reproducibility and relative validity of the short questionnaire to assess health-enhancing physical activity. *J Clin Epidemiol.* 2003;56(12):1163–1169

27. Bauer J, Capra S, Ferguson M. Use of the scored Patient-Generated Subjective Global Assessment (PG-SGA) as a nutrition assessment tool in patients with cancer. *Eur J Clin Nutr.* 2002;56(8):779–785

28. Cruz-Jentoft AJ, Bahat G, Bauer J, et al. Sarcopenia: revised European consensus on definition and diagnosis. *Age Ageing.* 2019;48(1):16–31

29. Sayer AA, Cruz-Jentoft A. Sarcopenia definition, diagnosis and treatment: consensus is growing. *Age Ageing.* 2022;51(10):afac220

30. Marjolein Visser, Laura A. Schaap, J.S.M. Hobbelen, Stany Perkisas, Walther M.W.J. Sipers. Sarcopenie: screening en diagnose. *Ned Tijdschr Geneeskd.* 2019;163(D3824)

31. Cederholm T, Jensen GL, Correia MITD, et al. GLIM criteria for the diagnosis of malnutrition - A consensus report from the global clinical nutrition community. *Clin Nutr.* 2019;38(1):1–9

32. Mijn Voedingscentrum.<https://www.voedingscentrum.nl/nl.aspx> Web site. <https://mijn.voedingscentrum.nl/>. Accessed May 9, 2025

33. Herdman M, Gudex C, Lloyd A, et al. Development and preliminary testing of the new five-level version of EQ-5D (EQ-5D-5L). *Qual Life Res.* 2011;20(10):1727–1736

34. Questionnaires for the measurement of costs in economic evaluations<https://www.imta.nl/> Web site. <https://www.imta.nl/questionnaires/>. Accessed 18 Feb, 2022

35. Bouwmans C, Krol M, Brouwer W, Severens JL, Koopmanschap MA, Hakkaart L. IMTA Productivity Cost Questionnaire (IPCQ). *Value Health.* 2014;17(7):A550

36. Oken MM, Creech RH, Tormey DC, et al. Toxicity and response criteria of the Eastern Cooperative Oncology Group. *Am J Clin Oncol.* 1982;5(6):649–655

37. Common Terminology Criteria
for Adverse Events (CTCAE) Version 5.0*U.S. Department of Health and Human Services.* 2017

38. Eisenhauer EA, Therasse P, Bogaerts J, et al. New response evaluation criteria in solid tumours: revised RECIST guideline (version 1.1). *Eur J Cancer.* 2009;45(2):228–247
